# Risk assessment of industrial chemicals towards salmon species amalgamating QSAR, q-RASAR, and ARKA framework

**DOI:** 10.1016/j.toxrep.2025.102017

**Published:** 2025-04-05

**Authors:** Prodipta Bhattacharyya, Shubha Das, Probir Kumar Ojha

**Affiliations:** Drug Discovery and Development Laboratory (DDD Lab), Department of Pharmaceutical Technology, Jadavpur University, Kolkata 700032, India

**Keywords:** Salmon species, Toxicity, QSAR, Stacking, Screening

## Abstract

The extensive use of industrial chemicals poses a serious threat to aquatic species such as the salmon species, which, when consumed, can affect human beings via their dietary intake. Salmon ﬁsh is a vital source of protein for maintaining human health. The present study aims to estimate the toxicity of diverse chemicals using *in silico*-based global model involving three different salmon species: *Salmo salar*, *Oncorhynchus kisutch*, and *Oncorhynchus tshawytscha* encompassing the toxicity endpoint median lethal concentration (LC_50_). Primarily, a quantitative structure-activity relationship (QSAR) model is developed using molecular descriptors. QSAR model descriptors are integrated with the similarity and error-based measures of read-across to develop the read-across structure-activity relationship (RASAR) model. Another emerging dimensionality reduction modeling algorithm, arithmetic residuals in *K*-groups analysis (ARKA) is employed to enhance the model’s degree of freedom. Model quality was improved by hybrid model development which combined the feature matrix of the QSAR model with those of the RASAR and ARKA descriptors. Finally, to attain more trustworthy results and address the limitations of individual models, a partial least square (PLS)-based stacking model is developed using the predicted response values of QSAR, RASAR, ARKA, and hybrid models as descriptors. The stacking model outperforms the quality of the individual models which is evident from the determination coefficient R^2^ (0.713), leave-one-out cross-validated correlation coefficient (Q^2^_LOO_:0.697), predictive R^2^ (Q^2^_F1__: 0.797),_ Q^2^_F2_ (0.795) and lower value of root mean square error of prediction RMSEp (0.652). Additionally, classification modelling was performed with the feature matrix of the QSAR model by employing both linear and non-linear approaches. The developed stacking model can thus be used in environmental risk assessment aiding in toxicity data-gap filling and design of safe and green chemicals.

## Highlights

• Developed a global stacking model to predict aquatic toxicity for salmon species.
• Combined QSAR, q-RASAR, ARKA, and hybrid models for improved predictive accuracy.
• Imperative structural fragments contributing to salmon toxicity are identified.
• Validated the model with experimental data from various salmon species.
• The model offers a wide applicability domain for accurate prediction of toxicity.

## Graphical Abstract

**Figure:**
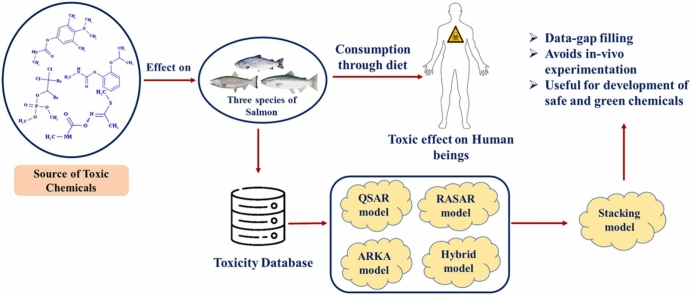

## 1 Introduction

Fish serve as a vital source of animal protein, offering significant nutritional and medicinal benefits to humans. However, with the rapid growth of the global population and industrial expansion, the demand for various chemicals has increased substantially. The widespread use of these chemicals in daily life raises serious concerns about their toxicological impact and environmental interactions. Industrialization and human activities have led to the contamination of water bodies, posing a severe threat to aquatic life. Hazardous chemicals, introduced into aquatic ecosystems through surface runoff, spray drift, and leaching from agricultural fields, accumulate in marine environments [1]. Additionally, the discharge of industrial and domestic wastewater into water bodies, further exacerbates the problem, endangering aquatic organisms and, in turn, human health through dietary exposure. Addressing these environmental challenges requires a deeper understanding of the toxicity of industrial chemicals and their long-term effects on aquatic species.

Salmon is one of the most widely consumed fish, with the largest consumers being the United States, the European Union, and Japan. They are euryhaline ray-finned fishes from the genera *Salmo* and *Oncorhynchus* of the Salmonidae family and are native to the tributaries of the North Atlantic (*Salmo*) and North Pacific (*Oncorhynchus*) basins [2]. As per the statistics of 2023, the global market size of salmon has reached 3.6 million tons [3]. Salmon is an excellent protein source and provides omega-3 fatty acids, reducing the risk of cardiovascular complications and mortality. Additionally, they can be used as therapeutic agents for rheumatoid arthritis [4] and possess considerable amounts of phosphorus, potassium, and vitamins such as B and D [5]. However, the increasing exposure of harmful chemicals to the marine environment through various industrial sources, pesticides, organic micro-pollutants, and radionuclides poses severe threats to these aquatic species. Various anthropogenic factors, such as hydropower regulation, habitat alteration, agricultural pollution, overexploitation, etc., have majorly resulted in the decline of Atlantic salmon due to their diadromous behavior [6]. Surface waters occupied by threatened and endangered salmon have been detected with malathion, which, at elevated water temperatures, increases the disease susceptibility and threatens both the aquatic and, consequently, terrestrial organisms [7]. Previous studies also reported the increasing concentration of organochlorine pesticides, dioxins, and mercury in water bodies, consequently affecting the salmon species. So an emerging concern has arisen to safeguard these species from the harmful effects of chemicals. Additionally, *in vitro* and *in vivo* methods of chemical toxicity testing often lead to the sacrifice of the species. So to address all the limitations associated with experimental testing and to protect the species from the effects of harmful chemicals, several *in silico* methods are being adopted [8], [9].

Several limitations are associated with the *in vitro* and *in vivo* methods for the evaluation of the aquatic toxicity profile of various chemicals. Various governing bodies have advised the use of *in silico* approaches such as quantitative structure-activity/property/toxicity relationship (QSAR/QSPR/QSTR), read-across (RA) and quantitative read-across structure-activity relationship (q-RASAR) to scrutinize the intrinsic properties concerning the prediction of the toxicity profile of a chemical [10]. QSAR aims to establish a mathematical correlation between the information obtained from a chemical constituent and the response, which may be activity/property/toxicity. Read-across, a non-statistical grouping approach based on the principle of molecular similarity, indicates that molecules with similar structural features are likely to have similar biological activity or properties. However, it is an unsupervised learning algorithm and cannot interpret essential features in most cases. The q-RASAR technique [11] is a supervised learning algorithm that enhances the model's predictive ability compared to conventional approaches. It mainly involves the integration of the structural and physicochemical descriptors of QSAR with the similarity and error-based measures of read-across. It usually produces simple, interpretable, reproducible models with enhanced predictivity. Recently, Banerjee and Roy developed a supervised dimensionality reduction technique for classification modeling known as the Arithmetic Residuals in *K*-groups Analysis (ARKA) [12], which has so far been applied for graded response data, aims to lessen the size of the descriptor matrix while retaining the appropriate chemical information. This may enhance a model's degree of freedom and statistical reliability. We have used this concept in our present work based on the regression modeling framework and attempted to explore its effects on quantitative experimental data. Various studies have been reported in recent years to evaluate the aquatic toxicity profile using *in silico* approaches. Gallagher et al. [13] used QSAR and q-RASAR approaches for toxicity assessment of various organic chemicals for *Labeo rohita*. Kumar et. al. [14] reported QSAR models encompassing multiple endpoints for assessing chronic aquatic toxicity of chemicals toward *Oryzias latipes*. Li et al. [15] reported QSTR models for assessing the ecotoxicological risk of pesticides to *Oncorhynchus mykiss*, *Colinus virginianus*, *Daphnia magna*, and rats. Chen et al. [16] developed QSAR models for fused/non-fused polycyclic aromatic hydrocarbons (FNFPAHs) toxicity towards *Pimephales promelas*. Khan et al. [17] used three different fish species for ecotoxicological risk assessment of 77 most-used pharmaceuticals during COVID-19. Yang et al. [18] performed chemometric modeling of three different species of tilapia against organic chemicals. Several multi-species toxicity prediction approaches utilizing machine learning techniques have been extensively explored in the literature. For instance, Ambure et al. [19] introduced QSAR-Co software (version 1.0.0), which facilitates the development of multitasking or multi-target classification-based QSAR models using linear discriminant analysis (LDA) and random forest (RF). Liu et al. [20] applied *in silico* methods to predict the aquatic toxicity of diverse chemicals across various crustacean species. Their study developed local binary models based on Mysidae data and global binary models incorporating data from Mysidae, Palaemonidae, and Penaeidae. These models were constructed using six machine learning algorithms: random forest (RF), naive Bayes (NB), k-nearest neighbor (kNN), C4.5 decision tree (CT), support vector machine (SVM), and artificial neural network (ANN). Furthermore, unsupervised machine learning techniques and graph theory have been employed to predict the acute eco-toxicity of chemical compounds [21]. Advancements in multi-target QSAR modeling have also been made through the development of QSAR-Co-X, a Python-based toolkit that integrates diverse chemical and biological information’s into a unified predictive framework [22]. Additionally, Halder et al. [23] proposed moving average multitasking models for assessing the eco-toxicity of endocrine-disrupting chemicals. A separate study introduced a quantitative multi-species toxicity modeling (qMTM) tool for predicting acute toxicity across algae, daphnia, and fish [24].

Beyond machine-learning-based QSAR models, other predictive approaches have utilized SMILES-based methods in conjunction with the Monte Carlo algorithm. These studies, leveraging the freely available CORAL software, have demonstrated notable success in predicting bioconcentration factors and lethal concentration values in fish species [25], [26]. While regression-based models remain widely used, classification-based QSAR models provide several advantages, including enhanced robustness and predictive accuracy, as demonstrated in various published studies [27], [28], [29]. Despite these advancements, limited studies have focused on toxicity prediction in salmon species. The present study addresses this gap by employing a global stacking ensemble model to predict the toxicity of diverse chemicals in salmon. By integrating multiple predictive approaches, the model enhances accuracy and generalizability, contributing valuable insights to the field of aquatic toxicology.

In the present study, we have developed global models combining the data points of three different species of salmon, namely *Salmo salar* (Atlantic salmon), *Oncorhynchus kisutch* (Silver salmon), and *Oncorhynchus tshawytscha* (Chinook salmon). We have combined the toxicity dataset for these three species of salmon and developed a global multispecies model. A global multispecies model has a wide domain of applicability, so it can assess the toxicity of multiple salmon fish species and eliminate the limitations of species-specific predictions. This helps reduce the use of resources such as time, cost, and modeling efforts. The endpoint used in this study was the negative log-transformed LC_50_, which is the amount of chemical inhaled by the test organism that causes death in 50 % of the population during the toxicity test study. We have employed several modeling algorithms, including QSAR, q-RASAR, and ARKA, strictly following the OECD guidelines. Further, to improve the quality and predictive ability of the established models, we have developed a hybrid model that combines the ARKA descriptors, RASAR descriptors, and the model descriptors of QSAR. Then, to address the limitations associated with individual models, we have developed a global stacking partial least squares (PLS) model using the fitted and predicted values of the training and test sets obtained from QSAR, q-RASAR, ARKA, and the hybrid model to define the feature matrix. The main idea of a “Global model” was to develop a model that can be universally treated as a reliable predictor for chemicals exerting toxicity to salmon. Model reliability, predictive ability, and interpretability were checked based on various internationally accepted validation parameters. Additionally, we have performed classification modelling with the feature matrix of the QSAR model by employing both linear and non-linear (random forest) approaches. The true predictive ability of the stacking PLS-based global model was evaluated by screening three other species of Pacific salmon, namely *Oncorhynchus nerka*, *Oncorhynchus keta*, and *Oncorhynchus gorbuscha*. The predicted toxicity value (pLC_50_) obtained from the global stacking model for these three species of salmon was validated to confirm model reliability. Screening of the pesticides properties database (PPDB) was also performed to further assess the external predictive ability and robustness of the model. Thus, our work aims for the eco-toxicological risk assessment of harmful chemicals, further aiding in data-gap filling and designing safer and eco-friendly chemicals.

## 2 Materials and method

### 2.1 Dataset

We have collected experimental toxicity data (LC_50_) of three different species of salmon, namely *Salmo salar*, *Oncorhynchus kisutch*, and *Oncorhynchus tshawytscha,* from the ECOTOX repository [30] and clubbed the individual datasets into a global dataset. Curation of the dataset was accomplished by removing the salts and metals. Then, we calculated the average of all the duplicate data points with numerical values close to each other and considered it a single data point. After curating the primary dataset, 106 compounds have been taken in the final dataset for further modeling. The two-dimensional (2D) structures of the chemicals were drawn using Marvin Sketch software [31] with the addition of explicit hydrogen atoms followed by ring aromatization. The collected experimental data points reported in active ingredient (AI) mg/L were converted to molar concentration and then transformed to a negative logarithmic scale (pLC_50_) to reduce the data range.

### 2.2 Descriptor computation and data pre-treatment

The numerical values associated with the chemical structure that establish a correlation with the response are known as descriptors. Zero to two-dimensional (0–2D) molecular descriptors were calculated using alvaDesc software [32]. It involved the computation of various descriptors based on structural and physicochemical parameters. From the initial pool, descriptors that are uninformative, inter-correlated (|r| > 0.95), and have constant (variance <0.0001) values were identified and removed using DataPreTreatmentGUI 1.2 software [33].

### 2.3 Dataset division

Dataset division, a vital step for model development, involves the division of the dataset into a training set and a test set [34]. The training set is involved in model development, and the test set is involved for validation purposes. Various approaches such as Kennard-Stone, Euclidean distance-based, activity-based, and modified *k*-Medoid clustering [33], [35] were involved in the current study. Among all the methods, the best outcomes were obtained in the activity-based division, with training and test sets composed of 80 and 26 compounds, respectively.

### 2.4 Feature selection and global QSAR model development

Feature selection is a fundamental step in QSAR model development. The predictivity and interpretability of the model are extremely attributed to the proper selection of the important and manageable number of descriptors contributing to the response. Feature selection removes irrelevant, insignificant, and noisy descriptors, thereby reducing the higher dimensional feature space to a lower dimensional feature space while retaining the important chemical information. A lower number of descriptors during QSAR modeling is attributed to more statistical significance [35]. With the help of a reduced descriptor matrix the risk of chance correlation and overfitting also reduces [36]. In this study, genetic algorithm (GA) was performed to identify significant descriptors [37]. The best subset selection (BSS) was performed to get a suitable combination of descriptors employing the BestSubsetSelectionModified_v2.1 tool [33]. A global QSAR model was developed by PLS regression using PLS_Single Y_version 1.0 software [38].

### 2.5 Read-across and calculation of the RASAR descriptors

Optimization of hyperparameters (number of close source compounds and similarity-based algorithm; σ, γ) from RA-based prediction is an essential pre-requisite for computing RASAR descriptors. The software Read-Across-v4.2.2 [39] involves the use of similarity-based methods such as Euclidean Distance (ED) based similarity, Gaussian Kernel (GK), and Laplacian Kernel (LK) function similarity for making predictions for the query set compounds [40], [41]. The training set of the QSAR model was divided into sub-train and sub-test sets, and hyperparameters were optimized using Auto_RA_Optimizer-v1.0 software [39]. The optimized setting was then used as an input criterion for predicting the original test set compounds. This optimized setting was further used for computing RASAR descriptors using RASAR-Desc-Calc-v3.0.3 software [39], where the training set descriptors are calculated by considering the training set to be the query set while the test set descriptors are computed by the test set as the query set. In both cases, the source/reference set was the training set.

### 2.6 Global q-RASAR model development

The set of 18 different RASAR descriptors were clubbed with the structural and physicochemical descriptors of QSAR, a process called data fusion [11], [42]. The pool of descriptors was then subjected to BSS method employing the BestSubsetSelectionModified_v2.1 tool [33]. A suitable descriptor combination was selected based on various validation metrics, which was then utilized to develop a global PLS-based q-RASAR model.

### 2.7 Global ARKA model development

QSAR models that are established using a higher number of modeling descriptors generally tend to be over-fitted, i.e., having a lower predictive performance for the test set and during cross-validation of the training set. On the other hand, developing models with a lower number of descriptors may result in the loss of chemical information. This has led to the development of an emerging dimensionality reduction technique known as the ARKA [12]. The size of the descriptor matrix is reduced in this approach, thus enhancing the degree of freedom. However, it retains the chemical information derived from the training set compounds. The point of difference from other dimensionality reduction methods like principal component analysis (PCA) and the t-distributed Stochastic Neighbor Embedding (t-SNE) is that ARKA uses a supervised algorithm. In contrast, PCA and t-SNE use an unsupervised approach. This enables ARKA to recognize activity cliffs and less confident data points. Activity cliffs refer to pairs or groups of structurally similar compounds that exhibit significantly different biological activities. These rapid changes in activity, despite minimal structural variations, pose challenges in predictive modeling, as small modifications in molecular structure can lead to extremely large differences in toxicity. The presence of activity cliffs can impact the reliability of QSAR models, often leading to decreased predictive accuracy and potential misclassification of toxic and non-toxic compounds. In toxicity prediction, ignoring activity cliffs can make models less accurate for similar compounds. To address this issue, strategies such as the identification and exclusion of activity cliffs from training datasets, the incorporation of advanced molecular descriptors that capture refined structural variations, and the use of similarity-based approaches have been proposed [43], [44]. These methods help to mitigate the effects of activity cliffs, improving model robustness and interpretability. As per the theory presented by Banerjee and Roy [12], the descriptor ARKA_1 encodes chemical information of the descriptors that have a higher discriminatory capacity towards the positive/active class. Similarly, the descriptor ARKA_2 encodes chemical information of the descriptors having a higher discriminatory ability for the negative/inactive class. Therefore, it should be expected that the positive/active compounds should have a positive value of ARKA_1 and a negative value of ARKA_2, and vice versa for the negative compounds. However, this is not always the case. In a simple 2D scatter plot of ARKA_2 (Y-axis) vs ARKA_1 (X-axis), it is expected that the positive compounds should ideally lie in the fourth quadrant (i.e. positive ARKA_1 and negative ARKA_2) and the negative compounds should ideally lie in the second quadrant (i.e. negative ARKA_1 and positive ARKA_2). However, there may be instances where a negative compound appears in the fourth quadrant and a positive compound appears in the second quadrant. Considering that these particular compounds do not lie close to either the axes (0.5 as threshold on either side on each axis, which is considered as a buffer zone), we can consider them as potential activity cliffs. The other data points lying in the first and third quadrants can be considered as less confident data points. This is an important diagnostic tool to assess the modelability of the dataset, prior to the deployment of mathematical modeling algorithms, where we can identify potential activity cliffs and less confident data points. In this work, we have checked the occurrence of activity cliffs in both our training and test sets using the ARKA_2 vs ARKA_1 plots. The ARKA descriptors were computed using the descriptors or the chemical information of the QSAR model. We have calculated ARKA descriptors for both the training and the test sets using a Java-based software ARKAdesc-v2.0 [45] and developed a regression-based model using MLRplusValidation1.3 software [46].

### 2.8 Hybrid model development

To improve the robustness and accuracy of the model, we have combined the six descriptors of the QSAR model, the 18 RASAR descriptors, and the 2 ARKA descriptors. This was subjected to the BSS method using the BestSubsetSelectionModified_v2.1 tool [33] to achieve a suitable descriptor combination. Further model was developed employing MLRplusValidation1.3 software [46].

### 2.9 PLS-based global stacking model development

Further, a global stacking model has been developed to attain more trustworthy, precise results and address the limitations associated with the individual models. Stacking is an approach to leverage the strengths of individual models and alleviate their limitations [47]. The stacking or meta-modeling approach incorporates the output from one modeling approach to be used as an input for another modeling algorithm. Here, we have combined the predictions obtained from the individual models mentioned above, i.e., forecasts from the QSAR model, RASAR model, ARKA model, and the hybrid model, which now serve as descriptors for the development of the stacking regressor model [48]. The final stacking model is developed using PLS_Single Y_version 1.0 software. The method of PLS regression aids in reducing the chance of inter-correlation between the independent variables. The main idea of PLS is the extraction of latent variables (LVs) which helps to reduce dimensionality and address multicollinearity, ultimately enhancing the robustness and interpretability of the model.

### 2.10 Classification-based QSAR modelling

We have developed Linear Discriminant Analysis (LDA) based classification model using a selected set of features or descriptors, which were previously employed in the QSAR model, and evaluated their predictive performance. The dataset was divided into two classes: toxic (pLC_50_ > 5.424) and non-toxic (pLC_50_ < 5.424), with the threshold determined based on the mean pLC_50_ value of the training set [49]. The dataset was splitted into training (70 %) and test (30 %) sets using Euclidean distance-based approach using QSAR-Co v.1.1.0 software [50] as this approach showed better statistical metrics than others. The GA framework incorporated the Matthews Correlation Coefficient (MCC) as the fitness function, with a constraint of six descriptors per equation. LDA was then applied using relevant descriptors, allowing the development of a classification model capable of distinguishing between toxic and non-toxic compounds. Internal validation of the model was conducted via cross-validation, while external validation was assessed using a separate test set. The model’s performance was evaluated using key statistical metrics, including accuracy, precision, sensitivity, specificity, F1-score, and MCC. Y-randomization test was carried out to assess the model’s robustness by comparing Wilk’s λ values between the original and randomized datasets [51]. In addition to the LDA-based classification model, a machine learning approach was implemented using a Random Forest (RF)-based classifier to check the non-linearity. The RF model was developed using the same QSAR-Co v.1.1.0 software, employing the same division and descriptor selection methodology (the following are the user-defined parameters for tuning the Random Forest model development, Each Bag Size =100, Maximum Depth = 0, number of randomly chosen features ‘n’ =0, Number of iterations =100, seed number (helps in regenerating the same Random Forest model =1), No. of folds (k) in cross-validation = 10). The model leveraged an ensemble of decision trees to enhance predictive performance, and hyperparameters were optimized using grid search. The final classification model was assessed based on cross-validation results, as well as external validation using the test set.

### 2.11 Statistical validation metrics

Rigorous validation of all the developed models was accomplished by employing various internationally recognized internal and external validation parameters to assess the predictivity and reliability of the models. Various internal validation metrics such as determination coefficient (R^2^) and cross-validated Q^2^_LOO_ are measures of goodness-of-fit and robustness of the model, respectively. R^2^ and Q^2^ have a threshold value of 0.6 and 0.5, respectively. External validation metrics such as Q^2^_F1_ and Q^2^_F2_, both with a threshold value of 0.5, are used to determine the model's predictive ability [52]. Model reliability was also assessed by calculating the concordance correlation coefficient (CCC), which measures both accuracy and precision and has a threshold value of 0.85. Mean Absolute Error (MAE) and root mean square error of prediction (RMSEp) were assessed, and a lower value suggests a strong and reliable model [35].

The mathematical formula for calculating all the calculated statistical metrics is shown in the equations below,

(1) $$R^{2}=1-\frac{\Sigma {\left(Y_{\mathrm{obs}\left(\mathrm{train}\right)}-Y_{\mathrm{calc}\left(\mathrm{train}\right)}\right)}^{2}}{\Sigma {\left(Y_{\mathrm{obs}\left(\mathrm{train}\right)}-{\bar{Y}}_{\mathrm{train}}\right)}^{2}}$$

(2) $$\mathrm{RMS}E_{p}=\sqrt{\frac{\Sigma {\left(Y_{\mathrm{obs}\left(\mathrm{test}\right)}-Y_{\mathrm{pred}\left(\mathrm{test}\right)}\right)}^{2}}{n}}$$

(3) $$Q_{\mathrm{LOO}}^{2}=1-\frac{\Sigma {\left(Y_{\mathrm{obs}\left(\mathrm{train}\right)}-Y_{\mathrm{pred}\left(\mathrm{train}\right)}\right)}^{2}}{\Sigma {\left(Y_{\mathrm{obs}\left(\mathrm{train}\right)}-{\bar{Y}}_{\mathrm{train}}\right)}^{2}}$$

(4) $$Q_{F1}^{2}=1-\frac{\Sigma {\left(Y_{\mathrm{obs}\left(\mathrm{test}\right)}-Y_{\mathrm{pred}\left(\mathrm{test}\right)}\right)}^{2}}{\Sigma {\left(Y_{\mathrm{obs}\left(\mathrm{test}\right)}-{\bar{Y}}_{\mathrm{train}}\right)}^{2}}$$

(5) $$Q_{F2}^{2}=1-\frac{\Sigma {\left(Y_{\mathrm{obs}\left(\mathrm{test}\right)}-Y_{\mathrm{pred}\left(\mathrm{test}\right)}\right)}^{2}}{\Sigma {\left(Y_{\mathrm{obs}\left(\mathrm{test}\right)}-{\bar{Y}}_{\mathrm{test}}\right)}^{2}}\;$$

(6) $$\mathrm{MAE}=\frac{\Sigma \left|Y_{\mathrm{obs}}-Y_{\mathrm{pred}}\right|}{n}$$

(7) $$\mathrm{CCC}=\frac{2\sum_{i=1}^{n}\left(x_{\mathrm{obs}\left(\mathrm{test}\right)}-\overset{®}{x_{\mathrm{obs}(\mathrm{test})}}\right)-\left(y_{\mathrm{pred}\left(\mathrm{test}\right)}-\overset{®}{y_{\mathrm{pred}(\mathrm{test})}}\right)}{\sum_{i=1}^{n}{\left(x_{\mathrm{obs}\left(\mathrm{test}\right)}-\overset{®}{x_{\mathrm{obs}(\mathrm{test})}}\right)}^{2}+\sum_{i=1}^{n}{\left(y_{\mathrm{pred}\left(\mathrm{test}\right)}-\overset{®}{y_{\mathrm{pred}(\mathrm{test})}}\right)}^{2}+n\left(\overset{®}{x_{\mathrm{obs}(\mathrm{test})}}-\overset{®}{y_{\mathrm{pred}(\mathrm{test})}}\right)}$$

Where,

$R^{2}=$ Determination coefficient

$Q_{\mathrm{LOO}}^{2}=$Leave-one-out cross-validated correlation coefficient

$Y_{\mathrm{obs}\left(\mathrm{train}\right)}=$Observed response values of the training set

$Y_{\mathrm{calc}\left(\mathrm{train}\right)}=$Calculated response values of the training set

$Y_{\mathrm{pred}\left(\mathrm{train}\right)}=$ Predicted response values of the training set

${\bar{Y}}_{\mathrm{train}}$=Average of all responses of the training set

$\mathrm{RMS}E_{P}=$Root mean square errors of prediction

n=Number of compounds

$Y_{\mathrm{obs}\left(\mathrm{test}\right)}=$Observed response values of the training set

$Y_{\mathrm{pred}\left(\mathrm{test}\right)}=\;$Predicted response values of the training set

${\bar{Y}}_{\mathrm{train}}$= Average of all responses of the training set

${\bar{Y}}_{\mathrm{test}}$= Average of all responses of the test set

$x_{\mathrm{obs}\left(\mathrm{test}\right)}$ = Observed response value of the test compound

$y_{\mathrm{pred}\left(\mathrm{test}\right)}$= Predicted response value of the test compound

$\overset{®}{x_{\mathrm{obs}(\mathrm{test})}}$= Average of the observed response value of the test compound

$\overset{®}{y_{\mathrm{pred}(\mathrm{test})}}$= Average of the predicted response value of the test compound

### 2.12 Y-randomization test

The Y-randomization test of the PLS-based global stacking model was also performed by employing SIMCA-P software version 16.0.2 [53] to check whether the model was developed by chance correlation. In randomization, the dependent variables are scrambled randomly while keeping the descriptor matrix constant, and new models are built. If the new randomly developed models have intercept values of R^2^_Y_ < 0.3 and Q^2^_Y_ < 0.05, suggesting that the developed model is not obtained by chance [54].

### 2.13 Applicability domain

Applicability domain (AD) is the theoretical space, knowledge, or domain in the chemical space surrounding both the response and the model descriptors. Prediction of the response value for the unknown/un-tested compounds should be performed only if the compounds lie within the domain of applicability [55]. AD study of the PLS-based global stacking model was performed with SIMCA-P software [52] using the DModX approach, maintaining a 99 % confidence level and a D-crit value of 0.009999.

### 2.14 Screening of external dataset

**Fig. 1:**
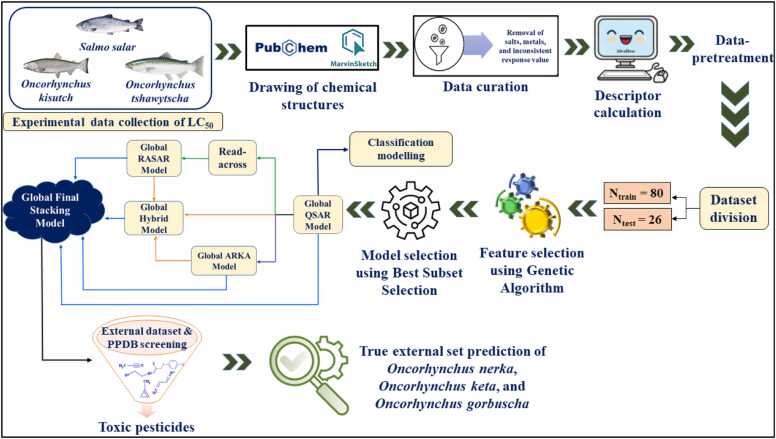
The workflow for developing the global PLS-based stacking model.

To assess the true predictive ability of the developed PLS-based global stacking model, we have used our model to make toxicity predictions for three other species of Pacific salmon, namely, *Oncorhynchus nerka*, *Oncorhynchus keta*, and *Oncorhynchus gorbuscha*. We have taken the external dataset for screening purposes from the literature [56]. Toxicity predictions using the PLS-based global stacking model have been performed for these three species separately, employing the Predictive Reliability Indicator (PRI tool_PLSVersion) [46] software. The reliability of predictions was assessed using AD, and the categorization of predictions was done as good, moderate, or bad [57]. Additionally, real-world validation using pesticides properties database (PPDB) data further supported the model’s predictive accuracy, as compounds predicted as toxic were also classified as toxic in experimental studies. The workflow for developing the global PLS-based stacking model is given in Fig. 1.

## 3 Results and discussion

A global PLS-based stacking model has been developed for the pLC_50_ toxicity endpoint by combining the data points of three different salmon species: *Salmo salar*, *Oncorhynchus kisutch*, and *Oncorhynchus tshawytscha*, strictly obeying the OECD guidelines. The training and test sets comprise 80 and 26 compounds, respectively.

### 3.1 Exploring the presence of activity cliffs in our dataset

**Fig. 2:**
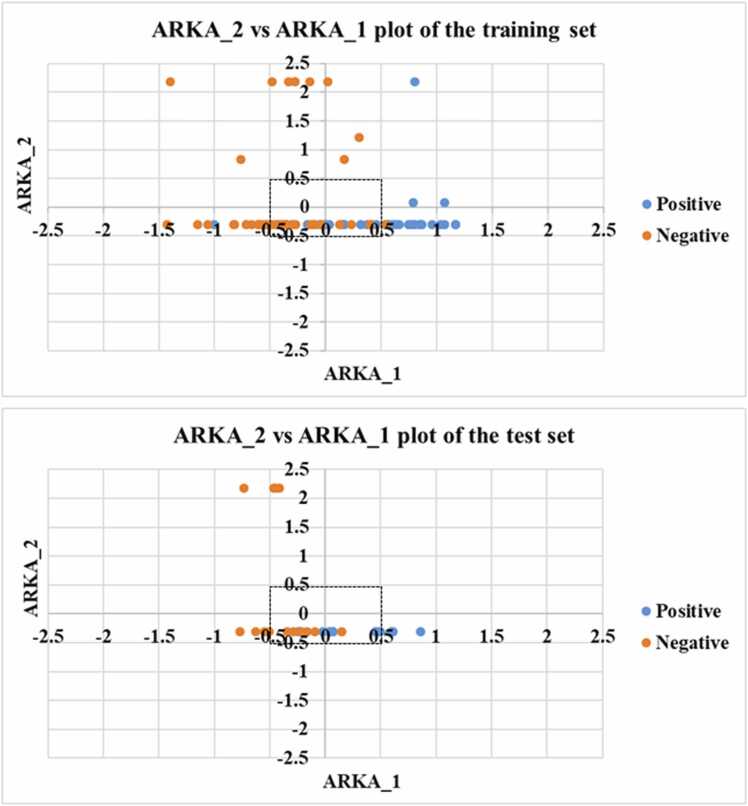
ARKA_2 vs ARKA_1 plots for the training and test sets.

Assessing the modelability of a given dataset is an important aspect that should be considered before the development of the mathematical models. In this study, we have used the novel supervised dimensionality reduction technique – the ARKA framework to assess the modelability and check the presence/absence of potential activity cliffs. Using the selected feature matrix of the QSAR model, we have computed the ARKA descriptors using the tool ARKAdesc-v2.0 [45]. We have then generated scatter plots of ARKA_2 vs ARKA_1 for the training and test sets. From the figure (Fig. 2), it can be observed that there were no potential activity cliffs due to the absence of confident data points in 2nd and 4th quadrants.

### 3.2 PLS-based global QSAR model

A global PLS-based QSAR model has been developed employing six descriptors with three latent variables (LVs). The equation for the PLS-based global QSAR model is given below in Eq. 8:

(8) $${\mathrm{pLC}}_{50}=\;3.51406-0.4447\times H-053+0.35901\times \mathrm{NsssCH}-1.10792\times B02\left[N-N\right]+2.13211\times B02\left[S-S\right]+0.57091\times B03\left[N-O\right]+0.46142\times \mathrm{LOGP}99\;$$

The model has been thoroughly corroborated with several internal and external validation parameters. The statistical metrics for the QSAR model are reported in Table 1**.** The values of R^2^ = 0.698 and Q^2^_LOO_ = 0.630, indicate the goodness-of-fit and robustness of the developed PLS-based QSAR model. Also, the value of Q^2^_F1_ = 0.773 and Q^2^_F2_ = 0.771 suggests the predictivity of the model.

**Table 1:** Statistical metrics of the developed models.

**Model details and validation metrics**	**Global QSAR model**	**Read-across**	**Global RASAR model**	**Global ARKA model**	**Global Hybrid model**	**Global PLS-based Stacking model**
**N**_**train**_**/N**_**test**_	80/26	-	80/26	80/26	80/26	**80/26**
**No. of descriptors**	6	-	6	2	5	**4**
**Number of LVs**	3	-	3	-	-	**1**
**R**^**2**^_**(train)**_	0.698	-	0.731	0.662	0.703	**0.713**
**Q**^**2**^_**LOO(train)**_	0.630	-	0.695	0.631	0.660	**0.697**
**MAE**_**LOO**_	0.770	-	0.701	0.748	0.714	**0.685**
**Q**^**2**^_**(F1)(test)**_	0.773	0.714	0.751	0.796	0.794	**0.797**
**Q**^**2**^_**(F2)(test)**_	0.771	0.711	0.748	0.794	0.792	**0.795**
**CCC**	0.853	-	0.848	0.868	0.874	**0.873**
**MAE**_**test**_	0.489	0.611	0.552	0.529	0.480	**0.490**
**RMSEp**	0.689	0.774	0.723	0.654	0.656	**0.652**

### 3.3 PLS-based global q-RASAR model

To improve the quality of the model, a q-RASAR model was developed using the same level of chemical information as used for QSAR model development. RASAR descriptors were computed based on the optimized hyperparameters setting obtained from read-across, including the Gaussian kernel setting with the number of similar training compounds = 10 and σ = 2. The merged descriptors pool (RASAR descriptors merged with the original QSAR descriptors) was subjected to the BSS method, and an appropriate combination of six descriptors was selected based on the statistical quality of the model. The q-RASAR model was developed using the PLS regression method, which comprised six descriptors with three latent variables. The equation for the PLS-based q-RASAR model is given below in Eq. 9:

(9) $${\mathrm{pLC}}_{50}=\;3.77066+0.37126\times \mathrm{NsssCH}+0.66907\times B02\left[S-S\right]+0.89969\times B03\left[N-O\right]+0.41638\times \mathrm{LOGP}99-2.5941\times \mathrm{SD similarity}\left(\mathrm{GK}\right)+1.10873\times s_{m}^{2}(\mathrm{GK})[\mathrm{Banerjee}-\mathrm{Roy similarity coefficient}2]\;$$

The statistical metrics for the q-RASAR model have been reported in Table 1**.** Here, it is observed that the values of R^2^ = 0.731 and Q^2^_LOO_ = 0.695, are significantly better than those of the PLS-based QSAR model.

We have developed dedicated software namely “SalTox-v1.0.” to facilitate the practical application of the q-RASAR model by regulatory agencies and industry stakeholders. The software provides a native interface for toxicity prediction, enabling users to input chemical structures and obtain toxicity predictions based on the developed q-RASAR model. A detailed user manual has been included namely “SalTox-v1.0.” in the Supplementary Information 2, outlining the software’s specifications, step-by-step instructions for running predictions, and guidance on interpreting results. SalTox-v1.0. is freely accessible for users from the following website (https://github.com/shubhamoy233/SalTox-v1.0.-software.git). By integrating the q-RASAR model into a user-friendly platform, we aim to enhance its accessibility and usability for risk assessment and regulatory decision-making. The software ensures reproducibility and ease of use, allowing seamless predictions of untested chemicals while maintaining model transparency and scientific rigor.

### 3.4 Global ARKA model

ARKA provides a supervised dimensionality reduction approach. The ARKA descriptors are calculated from the original QSAR descriptors and thus encode the selected feature space. The developed regression-based ARKA model is given below in Eq. 10:

(10) $${\mathrm{pLC}}_{50}=\;5.42393\;+1.88803\mathrm{ARKA\_}1\;-0.46825\mathrm{ARKA\_}2\;$$

The results for the regression-based ARKA model are given in Table 1. In the case of the ARKA model, the values of R^2^ = 0.662 and Q^2^_LOO_ = 0.631, are quite lower than that of both QSAR and RASAR models. However, the external predictive ability is enhanced as evidenced by the values of Q^2^_F1_ = 0.796 and Q^2^_F2_ = 0.794.

### 3.5 Hybrid model

A hybrid model was also developed to enhance the quality of the model. It involved the use of the six QSAR model descriptors, the eighteen RASAR descriptors, and the two ARKA descriptors. The equation for the hybrid model is given below in Eq. 11:

(11) pLC50=4.41194+1.03204B03[N−O]−0.10725g_m*Avg.Sim+1.15027Pos.Avg.Sim+0.42649s_m^2(GK)[Banerjee−Roy similarity coefficient2]+1.86677ARKA_1

The statistical metrics for the hybrid model are given in Table 1. The statistical metrics of the hybrid model showed that the internal and external validation performance are comparable to those of the other previously reported models in this study.

### 3.6 PLS-based global stacking model

The predictions obtained from the individual models, including the predictions from the QSAR model, RASAR model, ARKA model, and the hybrid model, were used as descriptors for developing the global PLS-based stacking regression model. This helps to address the limitations associated with the individual models, thus providing a more statistically robust and reliable model. The PLS-based global stacking model developed using one latent variable is given below in Eq. 12:

(12) $${\mathrm{pLC}}_{50}=\;-0.11205+0.25516\times \mathrm{QSARpred}+0.25517\times \mathrm{RASARpred}+0.25516\times \mathrm{ARKApred}+0.25516\times \mathrm{Hybridpred}$$

**Fig. 3:**
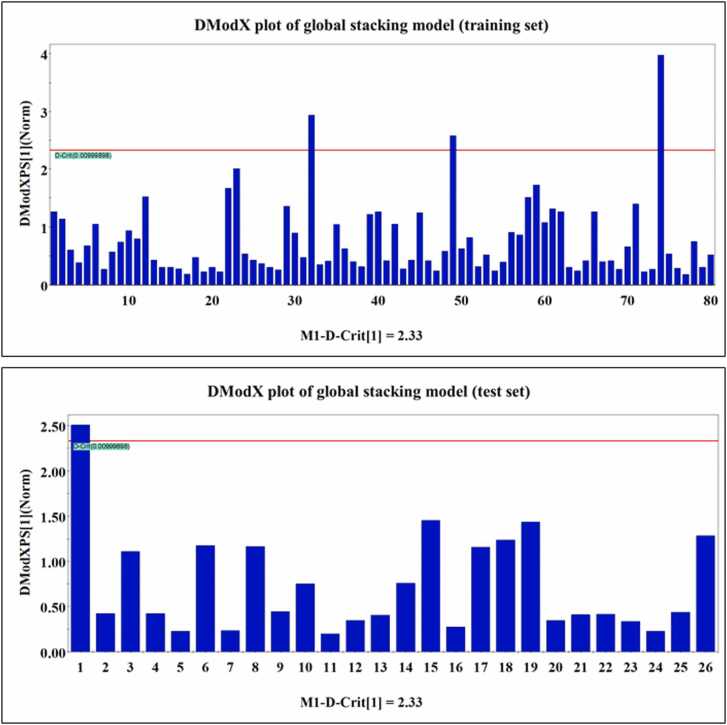
DModX plots for the training and test sets of PLS-based global stacking model.

**Fig. 4:**
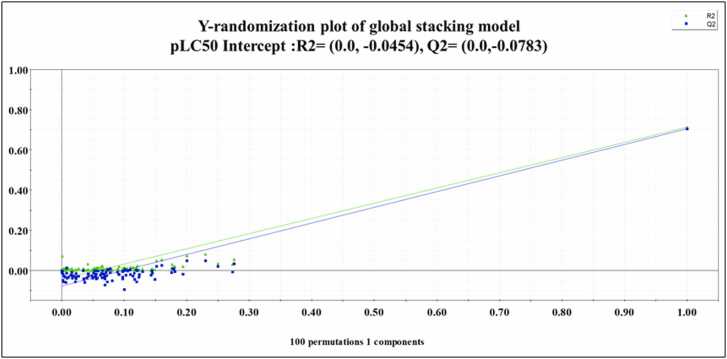
Y-randomization plot of PLS-based global stacking model.

**Fig. 5:**
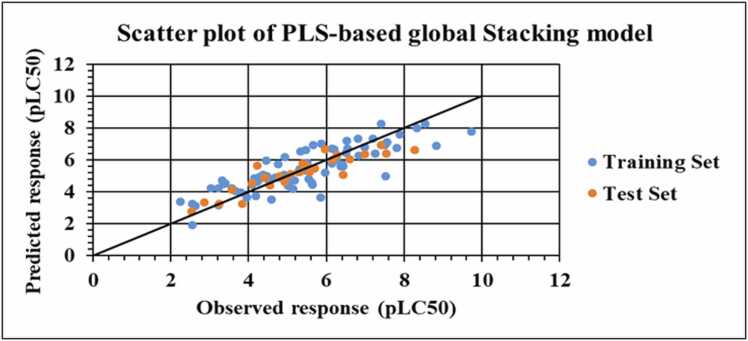
Scatter plot of PLS-based global stacking model.

The statistical metrics of the developed PLS-based global stacking model are given in Table 1. The reported validation metrics show that the stacking model outperforms the other individual models in terms of both the internal and external validation metrics. SIMCA-P software [58] generated various plots for the PLS-based global stacking model. Applicability domain assessment for both the training and the test sets has been performed using the DModX approach. DModX plots for both the training and test sets are provided in Fig. 3. The DModX plot of the PLS-based global stacking model suggests that **azadirachtin** (compound number 39), **antimycin A** (compound number 67) and **azinphos-methyl** (compound number 97) are the outliers from the training set due to distinct structural dissimilarity from others in the training set. In contrast, the DModX plot for the test set suggests that **2-(Thiocyanomethylthio)benzothiazole** (compound number **6**) is outside the domain of applicability. Also, the results obtained from the Y-randomization test, i.e., R^2^_Y_ = -0.0454 and Q^2^_Y_ = -0.0783, comply with the threshold limit of R^2^_Y_ < 0.3 and Q^2^_Y_ < 0.05, hence suggesting that the model has not been developed as a result of chance correlation. The Y-randomization plot for the PLS-based global stacking model is provided in Fig. 4. A scatter plot of the observed and the predicted response values along the X- and Y-axis, respectively, is shown in Fig. 5, representing uniform scattering for the stacked model.

### 3.7 Classification modelling

The classification-based models developed indicated (presented in Table S1 and Table S2 of Supplementary Information 2) that linear GA-LDA-based classification models exhibit stronger predictive performance than RF-based classification models leveraging nonlinear relationships. The developed models successfully distinguish between toxic and non-toxic compounds, as confirmed by internal and external validation metrics. RF-based model leveraging non-linear relationship showed overfitting towards the endpoint. A comparative analysis with existing multi-species toxicity prediction models suggests that GA-LDA based classification model offers robust classification capabilities while maintaining interpretability and reliability. The Y-randomization test was performed over 50 iterations for the LDA model, yielded an average Wilk’s λ value of 0.9187 (provided in Supplementary Information 1), significantly higher than the original model’s Wilk’s λ of 0.419. The LDA-based classification model is given in Eq. 13.

(13) $${\mathrm{pLC}}_{50}=\;-2.101+2.728*B02\left[S-S\right]+0.337*\mathrm{NsssCH}-0.586*H-053\;-1.050*B02\left[N-N\right]+0.001*B03\left[N-O\right]+0.568*\mathrm{LOGP}99$$

### 3.8 Mechanistic introspections

**Fig. 6:**
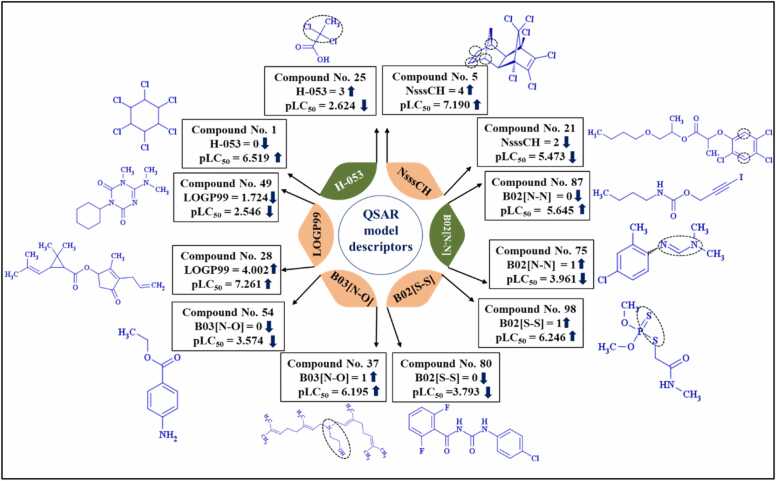
Insights into the contribution of the model descriptors using representative compounds.

As per OECD principle 5, a probable mechanistic interpretation has been provided. As all the individual models as well as the final stacking model are derived from the QSAR model, so we have provided an interpretation for the information’s obtained from QSAR model. A possible mechanistic introspection of the QSAR model is pictorially represented in Fig. 6. The descriptor **H-053** is an atom-centred fragment descriptor, which suggests that hydrogen atom attached to sp3 hybridized carbon atom with two halogen (X) atoms attached to the next carbon. The negative regression coefficient indicates that the presence this fragment leads to a decrease in toxicity towards the reference organism, as seen in **2,2-dichloropropanoic acid** (compound 25), while the absence of this fragment results in enhanced toxicity, as observed in **lindane** (compound 1) (provided in Fig. 6). The descriptor **NsssCH** is an atom-type E-state indices descriptor. This descriptor indicates the number of atoms of type sssCH, i.e. a carbon atom attached to one hydrogen atom and three other atoms with a single bond. The presence of three groups other than hydrogen results in increased bulkiness of the compound causing a decrease in the stability of the compound, making it more reactive and hence toxic. The positive regression coefficient confirms that this particular characteristic directly affects the response. A higher value of this characteristic increases the toxicity of compounds, as seen in the **aldrin** (compound 5). Conversely, a lower value of this characteristic reduces the toxicity of compounds, as demonstrated in the case of **kuran** (compound 21) (shown in Fig. 6). **B02[N-N]** is a 2D atom pair descriptor, which indicates the occurrence of two polar nitrogen atoms at topological distance 2 enhances the hydrophilicity of the compound [59], leading to the elimination of the compound readily from the body of the reference organism. This incidence is represented in compound **chlordimeform** (compound number 75), possessing this fragment shows a reduced toxicity value, while with the absence of this fragment, as depicted in the compound **iodopropynyl butylcarbamate** (compound number 87), a higher toxicity value is observed (provided in Fig. 6). **B02[S-S]** is a 2D atom pair descriptor which indicates the presence of electronegative atoms such as sulphur augments the overall electronegativity of the compound, which enhances the oxidative stress, further causing cell death [18]. This phenomenon is demonstrated in compound **dimethoate** (compound number 98), possessing this fragment, thus resulting in increased toxicity of the compound in the body of the reference organism. Conversely, the absence of this descriptor, as observed in the compound **diflubenzuron** (compound number 80), reduces the toxicity of the compound (demonstrated in Fig. 6). Another 2D atom pair descriptor, **B03[N-O]**, indicates the occurrence of electronegative atoms nitrogen and oxygen results in enhanced electronegativity of the compound. Also, an increase in electrostatic interactions is observed due to the presence of lone pair of electrons, thus enhancing the toxicity profile of the compound [14]. The positive regression coefficient of this descriptor signifies that the occurrence of this fragment in compounds such as **2-(Digeranylamino) ethanol** (compound number 37) is responsible for causing toxicity enhancement in the body of the reference organism. The absence of this fragment, as observed in **benzocaine** (compound number 54), results in a decreased toxicity profile of the compound (provided in Fig. 6). **LOGP99,** a descriptor of the type molecular property, indicates Wildmann-Crippen octanol-water partition coefficient (log P). It suggests that with the increase in the lipophilicity of the compound, it readily penetrates the blood-brain barrier (BBB) and other biological membranes of the reference organism, thus causing toxicity [14]. This is significant from **allethrin** (compound number 28), having a higher value of this descriptor, thus showing enhanced toxicity, while **hexazinone** (compound number 49), possessing a lower value of LOGP99, shows a reduction in the toxicity value (given in Fig. 6).

### 3.9 External dataset screening

The true external predictive ability of the global stacking model was assessed by screening the external datasets taken from literature [56] for three other species of Pacific salmon, including *Oncorhynchus nerka*, *Oncorhynchus keta*, and *Oncorhynchus gorbuscha*. This procedure was assisted by the PRI software [46]. The AD was assessed to check the reliability of the predicted values and it was found that all the data points fall within the applicability domain. The results obtained from the models were found to comply with real-world experimental data for most of the cases, thus suggesting that the established models are reliable and appropriate for making predictions about untested compounds for salmon species. The statistical results obtained from screening the external datasets using the global PLS-based stacking model is provided in Table 2**.** A strict comparison of the results obtained in the present study with previously reported studies could not be performed due to the different modeling algorithms employed and the altered arrangements of the training and test sets. In a recently published work, Yang et.al. [56] reported individual q-RASAR models for *Salmo salar*, *Oncorhynchus kisutch*, and *Oncorhynchus tshawytscha*. They also reported the QSAR model for *Oncorhynchus nerka*, *Oncorhynchus keta*, and *Oncorhynchus gorbuscha* species, using a small dataset modeling algorithm with a lower number of data points. Thus external predictive performance of these three species could not be obtained. Our study involves the development of a global PLS-based stacking model encompassing the *Salmo salar*, *Oncorhynchus kisutch*, and *Oncorhynchus tshawytscha* species. The global model developed thus helps to make toxicity predictions for *Oncorhynchus nerka*, *Oncorhynchus keta*, and *Oncorhynchus gorbuscha* species, with a lower prediction error. The developed PLS-based global stacking model enhances the applicability domain and addresses the limitations of species-specific toxicity predictions, by providing a reliable and broad-spectrum toxicity assessment. The results suggest that our model is reliable in making predictions for new/un-tested compounds and can be used for real-world applications. Additionally, the results obtained by screening the PPDB database showed that about 99.20 % of compounds lie within the AD with a good quality of prediction (approximately 99.93 % of compounds). The list of top and least 10 toxic compounds is given in Table 3 **(**reference for the same is provided in Supplementary Information 2**)**. The predictive results obtained were validated with real-world data suggesting that the global PLS-based stacking model can efficiently make accurate predictions and be used for toxicity data-gap filling. This multi-stage validation indicates the model's reliability and enhances the applicability of the model in the wide domain.

**Table 2:** Results of screening of the external dataset using PLS-based global stacking model.

**External validation metrics**	***Oncorhynchus gorbuscha***	***Oncorhynchus nerka***	***Oncorhynchus keta***
Q^2^_F1_	0.817	0.603	0.815
MAE	0.410	0.501	0.510
RMSEp	0.526	0.683	0.626

**Table 3:** List of top and least 10 toxic compounds of PPDB database.

**Sl. No.**	**Name of pesticide**	**Description**	**Reference**
**Top 10 toxic compounds of PPDB**			
1	Cholecalciferol	Toxic	I
2	Flucythrinate	It is extremely toxic to fish.	II
3	Fluvalinate	It is very highly toxic to fish.	III
4	Triacontanol	Toxic for fish and other aquatic animals.	IV
5	Acrinathrin	Toxic to most aquatic species.	V
6	Buthiobate	Low toxicity.	VI
7	Merphos	High fish acute eco-toxicity.	VII
8	Tribufos	Highly toxic to fish.	VIII
9	Difethialone	Highly toxic to birds and aquatic life.	IX
10	Cadusafos	Toxic to fish and aquatic invertebrates	X
**Least 10 toxic compounds of PPDB**			
1	Amitrole	Non-toxic to fish.	XI
2	Urea sulphate	Non-toxic to fish.	XII
3	Thiourea	Moderate to highly toxic in the aquatic compartment.	XIII
4	Ethylene urea	Low toxicity.	XIV
5	Cyanamide	Slightly toxic to fish.	XV
6	Mesosulfuron	Slightly toxic to fish.	XVI
7	Mesosulfuron-methyl	Slightly toxic to fish.	XVII
8	Dalapon	Low toxicity to fish.	XVIII
9	Azimsulfuron	Low toxicity towards fish.	XIX
10	Foramsulfuron	Non-toxic to fish.	XX

### 3.10 Scope and limitations of the study

In this study, we systematically explored different predictive models such as QSAR, q-RASAR, and ARKA, each identifying distinct sets of toxicity-relevant descriptors. These individual models provided complementary insights into the molecular determinants of toxicity. To leverage the strengths of each approach, we developed a hybrid model that integrated the most significant descriptors from all three models. Building upon this, we constructed a global stacking model that utilized the predictions from all four models (QSAR, q-RASAR, ARKA, and the hybrid model) as input features to generate a final toxicity prediction. This hierarchical approach allowed us to refine toxicity classification and improve predictive accuracy by combining the strengths of multiple modeling strategies. The stacking model’s ability to generate multiple predictive outputs aligns well with modern risk assessment methodologies. By incorporating its predictions into regulatory frameworks, toxicity assessments can achieve higher reliability, reducing uncertainties associated with single-model approaches. The integration of stacking model predictions into regulatory decision-making allows for a more comprehensive evaluation of chemical toxicity, ensuring a scientifically robust and data-driven approach for risk assessment.

The integration of stacking model predictions into risk assessment methodologies enhances the robustness of toxicity evaluations, reducing uncertainties associated with single-model predictions. By incorporating these advanced predictive insights into regulatory frameworks, chemical risk assessment can be conducted with greater confidence, ultimately improving decision-making processes for environmental and human health protection.

## 4 Conclusion

Salmon is a widely consumed fish and serves as a good source of protein and omega-3 fatty acids. The essence of this work lies in the direct linking of chemical toxicity associated with different species of salmon, which consequently affects human beings via dietary intake and causes environmental damage. The present study reports the first PLS-based global stacking model by combining the data points for three species of salmon, namely *Salmo salar*, *Oncorhynchus kisutch*, and *Oncorhynchus tshawytscha,* to estimate the toxicity of diverse chemicals. The global multispecies model can be universally treated as a reliable predictor for chemicals exerting toxicity to salmon. It has a wide domain of applicability, so it can assess the toxicity of multiple salmon fish species and eliminate the limitations of species-specific predictions. Here, we have developed a global QSAR model against multiple salmon species. From the QSAR model, it can be stated that the occurrence of the NsssCH fragment, the presence of electronegative atoms such as sulphur, nitrogen, oxygen, and increased lipophilicity of the compound results in the enhancement of the toxicological potential of the chemical in the body of the reference organism. While compounds with hydrogen atoms attached to sp3 hybridized carbon, which in turn is attached with two halogen (X) atoms to the next carbon, the presence of two polar nitrogen atoms increases the hydrophilicity of the compound, thus increasing their aqueous solubility and resulting in their easy elimination from the body of the reference organisms. Identifying these specific features helps develop green and environment-friendly chemicals adhering to the RRR principles (reduction, refinement, and replacement). We have also developed q-RASAR, ARKA, and hybrid models using same level of chemical information to enhance the robustness, quality, and predictivity of the model. To address the limitations associated with the individual models and to get more trustworthy results, we have developed a PLS-based global stacking model for the salmon fish using all the information of each individual model. The statistical metrics obtained from the PLS-based global stacking model suggest that it outperforms the quality of the individual models, confirming the model's robustness, predictive ability, and goodness of fit. Moreover, classification-based models were developed using the feature matrix of the QSAR model by employing both linear (GA-LDA) and non-linear (RF) approaches to distinguish between toxic and non-toxic compounds. Further, to assess the true external predictive ability of the PLS-based global stacking model, we have screened the external datasets for three other species of Pacific salmon, namely *Oncorhynchus nerka*, *Oncorhynchus keta*, and *Oncorhynchus gorbuscha*. Screening of the PPDB database was also carried out to identify the potential toxicants and check the robustness of the developed global stacking model. The prediction values obtained from the model comply with real-world experimental data. Overall, the development of a PLS-based global stacking model for evaluating the toxicity profile of diverse chemicals against multiple salmon species represents a trustworthy tool that supports regulatory decision-making and eventually contributes to a more sustainable environment.

## Funding sources

No specific funding has been received by the author(s) for this work.

## CRediT authorship contribution statement

**OJHA PROBIR KUMAR:** Writing – review & editing, Visualization, Supervision, Investigation, Conceptualization. **Das Shubha:** Writing – review & editing, Supervision, Methodology, Investigation, Data curation, Conceptualization. **Bhattacharyya Prodipta:** Writing – original draft, Visualization, Validation, Formal analysis, Data curation, Conceptualization.

## Declaration of Competing Interest

The authors declare that they have no known competing financial interests or personal relationships that could have appeared to influence the work reported in this paper.

## Data Availability

Data will be made available on request.

## Appendix A Supplementary material

The whole dataset with the chemical name, corresponding SMILES, and pLC_50_ values; The descriptors of the PLS-based QSAR model; The descriptors of the PLS-based q-RASAR model; The descriptors of the ARKA model; The descriptors of the hybrid model; The descriptors of the PLS-based global stacking model; Output file of screening for the external dataset of *Oncorhynchus nerka*, *Oncorhynchus keta*, and *Oncorhynchus gorbuscha*. Output file of screening of PPDB database; Dataset for the Classification model; Y-randomization for LDA model

**Table S1.** Statistical validation parameters of the developed LDA-based QSAR model; **Table S2.** Statistical validation parameters of the developed RF (random forest) LDA-based QSAR model; **Table S3.** List of top and least 10 toxic compounds of PPDB database; User manual for SalTox-v1.0

## Footnotes

Appendix A Supplementary data associated with this article can be found in the online version at doi:10.1016/j.toxrep.2025.102017.

## References

[1] Kumar R., Sankhla M.S., Kumar R., Sonone S.S. (2021). Impact of pesticide toxicity in aquatic environment. Biointerface Res. Appl. Chem..

[2] Behnke R. (2010).

[3] 〈https://www.imarcgroup.com/salmon-market〉, accessed on: 12/07/2024.

[4] Calder P.C., Yaqoob P. (2009). Understanding omega-3 polyunsaturated fatty acids. Postgrad. Med..

[5] Jensen I.J., Mæhre H.K., Tømmerås S., Eilertsen K.E., Olsen R.L., Elvevoll E.O. (2012). Farmed Atlantic salmon (Salmo salar L.) is a good source of long chain omega-3 fatty acids. Nutr. Bull..

[6] Forseth T., Barlaup B.T., Finstad B., Fiske P., Gjøsæter H., Falkegård M., Hindar A., Mo T.A., Rikardsen A.H., Thorstad E.B., Vøllestad L.A. (2017). The major threats to Atlantic salmon in Norway. ICES J. Mar. Sci..

[7] Dietrich J.P., Van Gaest A.L., Strickland S.A., Arkoosh M.R. (2014). The impact of temperature stress and pesticide exposure on mortality and disease susceptibility of endangered Pacific salmon. Chemosphere.

[8] Lundebye A.K., Lock E.J., Rasinger J.D., Nøstbakken O.J., Hannisdal R., Karlsbakk E., Wennevik V., Madhun A.S., Madsen L., Graff I.E., Ørnsrud R. (2017). Lower levels of persistent organic pollutants, metals and the marine omega 3-fatty acid DHA in farmed compared to wild Atlantic salmon (Salmo salar). Environ. Res..

[9] Nøstbakken O.J., Hove H.T., Duinker A., Lundebye A.K., Berntssen M.H., Hannisdal R., Lunestad B.T., Maage A., Madsen L., Torstensen B.E., Julshamn K. (2015). Contaminant levels in Norwegian farmed Atlantic salmon (Salmo salar) in the 13-year period from 1999 to 2011. Environ. Int..

[10] Nicolotti O., Benfenati E., Carotti A., Gadaleta D., Gissi A., Mangiatordi G.F., Novellino E. (2014). REACH and in silico methods: an attractive opportunity for medicinal chemists. Drug Discov. Today.

[11] Banerjee A., Roy K. (2022). First report of q-RASAR modeling toward an approach of easy interpretability and efficient transferability. Mol. Divers..

[12] Banerjee A., Roy K. (2024). ARKA: a framework of dimensionality reduction for machine-learning classification modeling, risk assessment, and data gap-filling of sparse environmental toxicity data. Environ. Sci.: Process. Impacts.

[13] Gallagher A., Kar S. (2024). Unveiling first report on in silico modeling of aquatic toxicity of organic chemicals to Labeo rohita (Rohu) employing QSAR and q-RASAR. Chemosphere.

[14] Kumar A., Ojha P.K., Roy K. (2024). Safer and greener chemicals for the aquatic ecosystem: Chemometric modeling of the prolonged and chronic aquatic toxicity of chemicals on Oryzias latipes. Aquat. Toxicol..

[15] Li Y., Fan T., Ren T., Zhang N., Zhao L., Zhong R., Sun G. (2024). Ecotoxicological risk assessment of pesticides against different aquatic and terrestrial species: using mechanistic QSTR and iQSTTR modeling approaches to fill the toxicity data gap. Green. Chem..

[16] Chen S., Sun G., Fan T., Li F., Xu Y., Zhang N., Zhao L., Zhong R. (2023). Ecotoxicological QSAR study of fused/non-fused polycyclic aromatic hydrocarbons (FNFPAHs): Assessment and priority ranking of the acute toxicity to Pimephales promelas by QSAR and consensus modeling methods. Sci. Total Environ..

[17] Khan K., Kar S., Roy K. (2023). Are we ready to combat the ecotoxicity of COVID-19 pharmaceuticals? An in silico aquatic risk assessment. Aquat. Toxicol..

[18] Yang S., Kar S. (2024). First report on chemometric modeling of tilapia fish aquatic toxicity to organic chemicals: Toxicity data gap filling. Sci. Total Environ..

[19] Ambure P., Halder A.K., Gonzalez Diaz H., Cordeiro M.N.D. (2019). QSAR-Co: an open source software for developing robust multitasking or multitarget classification-based QSAR models. J. Chem. Inf. Model..

[20] Liu L., Yang H., Cai Y., Cao Q., Sun L., Wang Z., Li W., Liu G., Lee P.W., Tang Y. (2019). In silico prediction of chemical aquatic toxicity for marine crustaceans via machine learning. Toxicol. Res..

[21] Takata M., Lin B.L., Xue M., Zushi Y., Terada A., Hosomi M. (2020). Predicting the acute ecotoxicity of chemical substances by machine learning using graph theory. Chemosphere.

[22] Halder A.K., Dias Soeiro Cordeiro M.N. (2021). QSAR-Co-X: an open source toolkit for multitarget QSAR modelling. J. Chemin.-..

[23] Halder A.K., Moura A.S., Cordeiro M.N.D. (2023). Predicting the ecotoxicity of endocrine disruptive chemicals: Multitasking in silico approaches towards global models. Sci. Total Environ..

[24] Gajewicz-Skretna A., Wyrzykowska E., Gromelski M. (2023). Quantitative multi-species toxicity modeling: Does a multi-species, machine learning model provide better performance than a single-species model for the evaluation of acute aquatic toxicity by organic pollutants?. Sci. Total Environ..

[25] Toropova A.P., Toropov A.A., Martyanov S.E., Benfenati E., Gini G., Leszczynska D., Leszczynski J. (2013). CORAL: Monte Carlo method as a tool for the prediction of the bioconcentration factor of industrial pollutants. Mol. Inform..

[26] Toropov A.A., Toropova A.P., Benfenati E. (2020). QSAR model for pesticides toxicity to Rainbow Trout based on “ideal correlations. Aquat. Toxicol..

[27] Ai H., Wu X., Zhang L., Qi M., Zhao Y., Zhao Q., Zhao J., Liu H. (2019). QSAR modelling study of the bioconcentration factor and toxicity of organic compounds to aquatic organisms using machine learning and ensemble methods. Ecotoxicol. Environ. Saf..

[28] Huang K., Zhang H. (2022). Classification and regression machine learning models for predicting aerobic ready and inherent biodegradation of organic chemicals in water. Environ. Sci. Technol..

[29] Qin L.T., Zhang J.Y., Nong Q.Y., Zeng H.H., Liang Y.P., Mo L.Y. (2024). Classification and regression machine learning models for predicting the combined toxicity and interactions of antibiotics and fungicides mixtures. Environ. Pollut..

[30] 〈https://cfpub.epa.gov/ecotox/〉 accessed on: 10/03/2024.

[31] 〈https://chemaxon.com/marvin〉 accessed on: 17/03/2024.

[32] Mauri A. (2020). alvaDesc: A tool to calculate and analyze molecular descriptors and fingerprints. Ecotoxicological QSARs.

[33] Ambure P., Aher R.B., Gajewicz A., Puzyn T., Roy K. (2015). NanoBRIDGES” software: open access tools to perform QSAR and nano-QSAR modeling. Chemom. Intell. Lab. Syst..

[34] Martin T.M., Harten P., Young D.M., Muratov E.N., Golbraikh A., Zhu H., Tropsha A. (2012). Does rational selection of training and test sets improve the outcome of QSAR modeling?. J. Chem. Inf. Model..

[35] Roy K., Kar S., Das R.N. (2015).

[36] Gonzalez M.P., Teran C., Saiz-Urra L., Teijeira M. (2008). Variable selection methods in QSAR: an overview. Curr. Top. Med. Chem..

[37] Goodarzi M., Dejaegher B., Heyden Y.V. (2012). Feature selection methods in QSAR studies. J. AOAC Int..

[38] Wold S., Sjöström M., Eriksson L. (2001). PLS-regression: a basic tool of chemometrics. Chemom. Intell. Lab. Syst..

[39] 〈https://sites.google.com/jadavpuruniversity.in/dtc-lab-software/home〉 accessed on:15/08/2024.

[40] Chatterjee M., Banerjee A., De P., Gajewicz-Skretna A., Roy K. (2022). A novel quantitative read-across tool designed purposefully to fill the existing gaps in nanosafety data. Environ. Sci.: Nano.

[41] Das S., Samal A., Ojha P.K. (2024). Chemometrics-driven prediction and prioritization of diverse pesticides on chickens for addressing hazardous effects on public health. J. Hazard. Mater..

[42] Banerjee A., Kar S., Pore S., Roy K. (2023). Efficient predictions of cytotoxicity of TiO2-based multi-component nanoparticles using a machine learning-based q-RASAR approach. Nanotoxicology.

[43] Cruz-Monteagudo M., Medina-Franco J.L., Pérez-Castillo Y., Nicolotti O., Cordeiro M.N.D., Borges F. (2014). Activity cliffs in drug discovery: Dr Jekyll or Mr Hyde?. Drug Discov. Today.

[44] Schür C., Gasser L., Perez-Cruz F., Schirmer K., Baity-Jesi M. (2023). A benchmark dataset for machine learning in ecotoxicology. Sci. Data.

[45] 〈https://sites.google.com/jadavpuruniversity.in/dtc-lab-software/arithmetic-residuals-in-k-groups-analysis-arka〉 accessed on: 20/10/2024.

[46] 〈https://teqip.jdvu.ac.in/QSAR_Tools/〉 accessed on: 14/05/2024.

[47] Yan X., Yue T., Winkler D.A., Yin Y., Zhu H., Jiang G., Yan B. (2023). Converting nanotoxicity data to information using artificial intelligence and simulation. Chem. Rev..

[48] Varsou D.D., Banerjee A., Roy J., Roy K., Savvas G., Sarimveis H., Wyrzykowska E., Balicki M., Puzyn T., Melagraki G., Lynch I. (2024). The Round Robin approach applied to nanoinformatics: consensus prediction of nanomaterials zeta potential. Beilstein Arch..

[49] Das S., Bhattacharjee A., Ojha P.K. (2025). First report on q-RASTR modelling of hazardous dose (HD5) for acute toxicity of pesticides: an efficient and reliable approach towards safeguarding the sensitive avian species. SAR QSAR Environ. Res..

[50] Ambure P., Halder A.K., Gonzalez Diaz H., Cordeiro M.N.D. (2019). QSAR-Co: an open source software for developing robust multitasking or multitarget classification-based QSAR models. J. Chem. Inf. Model..

[51] Guha R., Jurs P.C. (2005). Determining the validity of a QSAR model− a classification approach. J. Chem. Inf. Model..

[52] De P., Kar S., Ambure P., Roy K. (2022). Prediction reliability of QSAR models: an overview of various validation tools. Arch. Toxicol..

[53] 〈https://www.sartorius.com/en/products/process-analytical-technology/data-analytics-software/mvda-software/simca〉. accessed on: 05/11/2024.

[54] Rücker C., Rücker G., Meringer M. (2007). y-Randomization and its variants in QSPR/QSAR. J. Chem. Inf. Model..

[55] Mukherjee R.K., Kumar V., Roy K. (2021). Ecotoxicological QSTR and QSTTR modeling for the prediction of acute oral toxicity of pesticides against multiple avian species. Environ. Sci. Technol..

[56] Yang S., Kar S. (2024). How safe are wild-caught salmons exposed to various industrial chemicals? First ever in silico models for salmon toxicity data gaps filling. J. Hazard. Mater..

[57] Roy K., Ambure P., Kar S. (2018). How precise are our quantitative structure–activity relationship derived predictions for new query chemicals?. ACS Omega.

[58] Wu Z., Li D., Meng J., Wang H. (2010). Introduction to SIMCA-P and its application. Handb. Partial least Sq.: Concepts, Methods Appl..

[59] Roy J., Ghosh S., Ojha P.K., Roy K. (2019). Predictive quantitative structure–property relationship (QSPR) modeling for adsorption of organic pollutants by carbon nanotubes (CNTs). Environ. Sci.: Nano.

