# Supplementary material for: Risk assessment of industrial chemicals towards salmon species amalgamating QSAR, q-RASAR, and ARKA framework
Source: Toxicol Rep. 2025 Apr 5;14:102017. doi: 10.1016/j.toxrep.2025.102017 (PMC12008129; doi:10.1016/j.toxrep.2025.102017)
Supplement: Supplementary file 2 — Table S1. Statistical validation parameters of the developed LDA-based QSAR model; Table S2. Statistical validation parameters of the developed RF (random forest) LDA-based QSAR model; Table S3. List of top and least 10 toxic compounds of PPDB database; User manual for SalTox-v1.0 [file mmc2.docx]

**Risk Assessment of Industrial Chemicals Towards Salmon Species Amalgamating QSAR, q-RASAR, and ARKA Framework**

Prodipta Bhattacharyya, Shubha Das, Probir Kumar Ojha*

Drug Discovery and Development Laboratory (DDD Lab),

Department of Pharmaceutical Technology, Jadavpur University,

Kolkata 700032, India

*Corresponding author: P K Ojha, Email: [probirojha@yahoo.co.in](mailto:probirojha@yahoo.co.in), [pkojha.pharmacy@jadavpuruniversity.in](mailto:pkojha.pharmacy@jadavpuruniversity.in)

ORCID id: 0000-0003-4796-3915, Phone: +91 8777677004

**Contents:**

1. **Table S1.** Statistical validation parameters of the developed LDA-based QSAR model.
2. **Table S2.** Statistical validation parameters of the developed RF (random forest) LDA-based QSAR model.
3. **Table S3:** List of top and least 10 toxic compounds of PPDB.
4. SalTox-v1.0.

**Table S1.** Statistical validation parameters of the developed LDA-based QSAR model.

| *Metrics* | *Internal validation*  *(training set)* | *External validation*  *(test set)* |
| --- | --- | --- |
| No. of samples | 75 | 31 |
| Accuracy (in %) | 89.33 | 80.64 |
| Precision (in %) | 93.75 | 82.35 |
| Sensitivity (in %) | 83.33 | 82.35 |
| Specificity (in %) | 94.87 | 78.57 |
| F-measure | 0.882 | 0.823 |
| MCC | 0.79 | 0.609 |

**Table S2.** Statistical validation parameters of the developed RF (random forest) LDA-based QSAR model.

| *Metrics* | *Internal validation*  *(training set)* | *External validation*  *(test set)* |
| --- | --- | --- |
| No. of samples | 75 | 31 |
| Accuracy (in %) | 100 | 80.645 |
| Precision (in %) | 100 | 61.538 |
| Sensitivity (in %) | 100 | 88.88 |
| Specificity (in %) | 100 | 77.27 |
| F-measure | 1 | 0.727 |
| MCC | 1 | 0.6086 |

**Table S3:** List of top and least 10 toxic compounds of PPDB.

| **Sl. No.** | **Name of pesticide** | **Description** | **Reference** |
| --- | --- | --- | --- |
| **Top 10 toxic compounds of PPDB** | | | |
| 1 | Cholecalciferol | Toxic | <https://www.aquafeed.com/newsroom/news/eu-vitamin-d3-safe-and-effective-for-fish-conditions-apply/> accessed on: 21/02/2025 |
| 2 | Flucythrinate | It is extremely toxic to fish. | <https://www.coastalwiki.org/wiki/Flucythrinate> accessed on: 21/02/2025 |
| 3 | Fluvalinate | It is very highly toxic to fish. | [https://extoxnet.orst.edu/pips/fluvalin.htm#](https://extoxnet.orst.edu/pips/fluvalin.htm) accessed on: 21/02/2025 |
| 4 | Triacontanol | Toxic for fish and other aquatic animals. | <https://ppqs.gov.in/sites/default/files/triacontanol_0.05_ec93fdow_agrosciences_india_p_ltd_1.pdf> accessed on: 21/02/2025 |
| 5 | Acrinathrin | Toxic to most aquatic species. | <http://sitem.herts.ac.uk/aeru/ppdb/en/Reports/16.htm#:~:text=It%20has%20a%20low%20toxicity,toxic%20to%20birds%20and%20earthworms>. accessed on: 21/02/2025 |
| 6 | Buthiobate | Low toxicity. | <http://sitem.herts.ac.uk/aeru/ppdb/en/Reports/2646.htm> accessed on: 21/02/2025 |
| 7 | Merphos | High fish acute eco-toxicity. | <http://sitem.herts.ac.uk/aeru/ppdb/en/Reports/440.htm> accessed on: 21/02/2025 |
| 8 | Tribufos | Highly toxic to fish. | <https://www3.epa.gov/pesticides/endanger/litstatus/effects/redleg-frog/tribufos/determination.pdf> accessed on: 21/02/2025 |
| 9 | Difethialone | Highly toxic to birds and aquatic life. | <http://sitem.herts.ac.uk/aeru/ppdb/en/Reports/233.htm#:~:text=It%20is%20highly%20toxic%20to,to%20birds%20and%20aquatic%20life>. accessed on: 21/02/2025 |
| 10 | Cadusafos | Toxic to fish and aquatic invertebrates | <https://en.wikipedia.org/wiki/Cadusafos#:~:text=Cadusafos%20has%20been%20proved%20to,bees%2C%20earthworms%20and%20other%20arthropods>. accessed on: 21/02/2025 |
| **Least 10 toxic compounds of PPDB** | | | |
| 1 | Amitrole | Non-toxic to fish. | <https://www3.epa.gov/pesticides/chem_search/reg_actions/reregistration/fs_PC-004401_1-Sep-96.pdf> accessed on: 21/02/2025 |
| 2 | Urea sulphate | Non-toxic to fish. | <https://downloads.regulations.gov/EPA-HQ-OPP-2010-0650-0002/content.pdf> accessed on: 21/02/2025 |
| 3 | Thiourea | Moderate to highly toxic in the aquatic compartment. | <https://www.inchem.org/documents/cicads/cicads/cicad49.htm> accessed on: 21/02/2025 |
| 4 | Ethylene urea | Low toxicity. | <http://sitem.herts.ac.uk/aeru/ppdb/en/Reports/874.htm> accessed on: 21/02/2025 |
| 5 | Cyanamide | Slightly toxic to fish. | <https://www3.epa.gov/pesticides/chem_search/cleared_reviews/csr_PC-014002_14-Sep-07_a.pdf> accessed on: 21/02/2025 |
| 6 | Mesosulfuron | Slightly toxic to fish. | <https://www3.epa.gov/pesticides/chem_search/reg_actions/registration/fs_PC-122009_31-Mar-04.pdf> accessed on: 21/02/2025 |
| 7 | Mesosulfuron-methyl | Slightly toxic to fish. | <https://www3.epa.gov/pesticides/chem_search/reg_actions/registration/fs_PC-122009_31-Mar-04.pdf> accessed on: 21/02/2025 |
| 8 | Dalapon | Low toxicity to fish. | <http://sitem.herts.ac.uk/aeru/ppdb/en/Reports/1613.htm#:~:text=It%20is%20highly%20soluble%20in,is%20moderately%20toxic%20to%20honeybees>. accessed on: 21/02/2025 |
| 9 | Azimsulfuron | Low toxicity towards fish. | <http://sitem.herts.ac.uk/aeru/ppdb/en/Reports/49.htm> accessed on: 21/02/2025 |
| 10 | Foramsulfuron | Non-toxic to fish. | <https://www3.epa.gov/pesticides/chem_search/reg_actions/registration/fs_PC-122020_27-Mar-02.pdf> accessed on: 21/02/2025 |

**SalTox-v1.0.**

**Overview:** The software provides a native interface for toxicity prediction towards salmon species, enabling users to obtain toxicity predictions based on the developed q-RASAR model.

**System requirement for using the software**

1. The user must download and install Python on their system before running the software.

2. There is a ‘dependencies’ file. Run one time that file in Python.

**Input file specifications**

The tool takes an input file having .xlsx extension containing the required set of descriptors for the query or the external set compounds to compute the median lethal toxicity of salmon species in terms of pLC_50_.

The query or the external set file should contain the compound number (No.) in the first column and the descriptors in the subsequent columns. **The user needs to maintain the same order of descriptors as shown in the figure.** The two-dimensional (2D) descriptors can be computed using alvaDesc software and the RASAR descriptors can be computed from RASAR-Desc-Calc-v3.0.3 software [1] with the optimized hyper-parameters being **Gaussian kernel setting with the number of similar training compounds = 10 and σ = 2.**


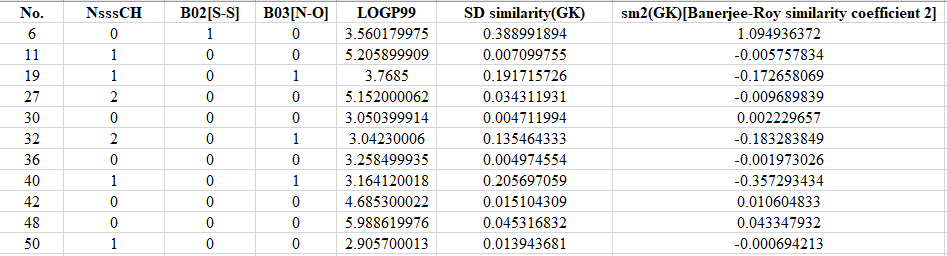


**Output file**

An excel **(.xlsx)** sheet named “**Predictions.xlsx**” is generated representing the predicted values of pLC_50_, as shown in the following figure.


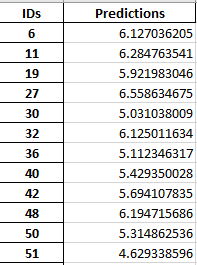


**Reference**

1. <https://sites.google.com/jadavpuruniversity.in/dtc-lab-software/home>
